# Diagnostic performance of glomerular PLA2R and THSD7A antibodies in biopsy confirmed primary membranous nephropathy in South Africans

**DOI:** 10.1186/s12882-020-02216-7

**Published:** 2021-01-07

**Authors:** Bingileki F Lwezaula, Oluwatoyin I Ameh, Udeme E Ekrikpo, Francois CJ Botha, Ugochi S Okpechi-Samuel, Nicola Wearne, Pierre Ronco, Aminu K Bello, Ikechi G. Okpechi

**Affiliations:** 1 Division of Nephrology and hypertension, University of Cape Town, Cape Town, South Africa; 2 Kidney and Hypertension Research Unit, University of Cape Town, Cape Town, South Africa; 3 Mount Meru Regional Referral Hospital, Arusha, Tanzania; 4 Division of Nephrology, Zenith Medical & Kidney Centre, Gudu, Abuja Nigeria; 5 Department of Medicine, University of Uyo, Uyo, Nigeria; 6 Pathcare Laboratories, George, South Africa; 7 Division of Anatomical pathology, University of Cape Town, Cape Town, South Africa; 8 Department of Internal Medicine, Federal Medical Centre, Jabi, Abuja, Nigeria; 9 Department of Nephrology and Dialysis, Assistance Publique-Hôpitaux de Paris, Tenon Hospital, Paris, France; 10 Department of Medicine, University of Alberta, Edmonton, Canada

**Keywords:** Africans, Primary membranous nephropathy, Lupus nephritis, PLA2R, THSD7A

## Abstract

**Background:**

Serum and tissue-based tests using phospholipase A2 receptor 1 (PLA2R) and thrombospondin type-1 domain containing 7A (THSD7A) are established immune biomarkers for the diagnosis of primary membranous nephropathy (PMN). This study assessed the diagnostic performance of these biomarkers in the diagnosis of PMN in South Africans.

**Methods:**

This was a cross-sectional analysis from a single centre in Cape Town, South Africa. Relevant biodata was collected from all patients. Histology, including slides for PLA2R and THSD7A were processed and assessed by typical microscopic and immunohistochemical features. Biopsy tissues of patients with membranous lupus nephritis (LN-V) and diabetic nephropathy (DN) were used as controls. The diagnostic accuracy for diagnosis of PMN using positive PLA2R and THSD7A were evaluated.

**Results:**

Of the 88 patients included, 41 had PMN with a mean age of 44.5 ± 17.5 years and 61.0% were female. Histologically, PLA2R and THSD7A were only positive in the PMN group (51.2% and 4.9%, respectively) but negative in both control groups. The sensitivity of PLA2R and THSD7A for identifying PMN was 51.2% and 4.9%, respectively. The sensitivity of both tests together was 53.7% while the specificity and positive predictive values (PPV) for any of the tests (alone or in combination) was 100%. There was no difference in the sensitivity and specificity when using PLA2R alone compared to combining the two tests (*p*=0.32).

**Conclusion:**

Glomerular staining of PLA2R and THSD7A could have potential diagnostic values in South Africans. This has implications on how immunotherapies can be initiated and used in these settings.

## Background

Membranous glomerulonephritis (MGN) is a leading cause of the nephrotic syndrome in adults and occurs as a primary disorder (primary membranous nephropathy – PMN) or secondary to other disorders like lupus nephritis, infections and certain drugs.[1–6] Primary membranous nephropathy is a kidney-specific autoimmune disorder in which autoantibodies develop against self, podocyte antigens.[5] Phospholipase A2 receptor 1 (PLA2R) and thrombospondin type-1 domain containing 7A (THSD7A) are two podocyte specific antigens that have been identified in PMN.[7, 8] Determining the presence or absence of PLA2R and anti-THSD7A antibodies in serum and renal biopsy specimen, has significantly reshaped the diagnostic approach to PMN by providing important immunologic information which aid in discriminating between primary and secondary MGN, as well as predicting clinical outcomes in PMN.[9] Clinically, PLA2R and anti-THSD7A antigens may be identified in sera or by immunostaining methods in renal biopsy samples. While quantitative and semi-quantitative serologic assays are now commercially available for PLA2R and THSD7A antibodies, it has been noted that a significant number of individuals with histologically confirmed antigens to PLA2R in glomerular deposits (and hence a biopsy diagnosis of PMN) have negative PLA2R serology results.[10] It has also been shown that early in the time course of PMN, due to delayed seroconversion, PLA2R may be negative in serum but demonstrable in histologic specimen.[11] Kidney biopsy tissue-based testing may thus be a veritable diagnostic tool in appropriately identifying PMN.

The sensitivity and specificity of these tissue-based antibody tests in identifying PMN have largely varied across studies. However, one meta-analysis reported a pooled sensitivity and specificity of 65% (63–67%) and 97% (97–98%) for serum PLA2R antibodies and 79% (76–81%) and 90% (88–92%), respectively, for glomerular PLA2R antigens.[12] Hoxha et al.,[13] in a prospective cohort of 345 patients with MN reported the prevalence of THSD7A-associated MN to be 2.6% with 92% sensitivity and a 100% specificity using immunofluorescence techniques. Given that MGN (both primary and secondary types due to hepatitis B and lupus nephritis) has been shown to be a common cause of nephrotic syndrome in adults in African studies of kidney biopsies, differentiating primary from secondary type is important for making therapeutic decisions.[3, 6, 14] There are currently no studies in Africa assessing the utility of serum or glomerular PLA2R or THSD7A for accurately identifying patients with PMN. The aim of this study is to assess the sensitivity and specificity of PLA2R and THSD7A for identifying PMN in South African patients using kidney biopsy tissue-based antibody tests.

## Methods

### Study population and study design

The study was approved by the Human Research Ethics Committee of the University of Cape Town (HREC/REF: 662/2017). This was a cross-sectional study of patients with native kidney biopsy proven PMN, collected consecutively over a 10-year period (2007 to 2016). Patients known with lupus nephritis (LN) with biopsy proven secondary membranous LN (LN-V) and patients with diabetes mellitus whose biopsies showed classical features of diabetic nephropathy (DN) were included as controls. All the patients were selected from the renal biopsy registry of the Division of Nephrology and Hypertension, Groote Schuur Hospital (GSH), University of Cape Town. Given the retrospective nature of this study, patient consent was not required for this study.

### Data collection

We collected relevant demographic, clinical, serological, biochemical and histological data of all patients. Data recorded were age, gender, race, systolic and diastolic blood pressures (SBP and DBP) and presence of oedema. Serological data collected include full blood count, serum creatinine, estimated glomerular filtration rate (eGFR) using the modification of diets in renal disease (MDRD) equation,[15] urine protein-creatinine ratio (UPCR), serum cholesterol and albumin. Serologies for human immunodeficiency virus (HIV), Hepatitis B, syphilis and anti-nuclear antibodies (ANA) and anti-double stranded deoxyribonucleic acid (anti-dsDNA) were checked in all patients to ensure that common secondary causes of membranous nephropathy were excluded.

### Histology

The diagnosis of PMN was based on typical light microscopy and immunohistochemistry (IHC) findings, showing thickening of glomerular basement membrane with or without spikes and granular deposition of immunoglobulin (Ig)G and C3 along the glomerular capillary walls.[16] All patients who had been labelled as PMN had tested negative for HIV, Hepatitis B and C, syphilis and for all immunological markers of lupus. For the histology, we collected data on number of glomeruli in each biopsy, presence of tubular atrophy, interstitial fibrosis and immunoglobulin deposits in IHC stains (IgG, IgA, IgM, C3) as well as the results for PLA2R and THSD7A. All histology results were entered as positive (present) or negative (absent). All slides for PLA2R and THSD7A were stained using standard methods as described by manufacturers and all were analyzed and reviewed by a single pathologist (FCJB). For the IHC staining, we utilized methods previously described in another study.[17] Briefly, 3 µm of formalin fixed paraffin embedded sections of biopsy specimen was deparaffinized and rehydrated. After endogenous peroxidase blockage and antigen retrieval, slides were incubated with the antibody. For the PLA2R stain, the antibody used was HPA012657-100UL (Sigma Aldrich, rabbit polyclonal antibody, dilution 1:1000) and for THSD7A, it was HPA000923-100UL (Sigma Aldrich, rabbit polyclonal antibody, dilution 1:1000). Multiple washes were performed, and the specimens were washed in phosphate buffered saline (PBS) and developed in 3,3′-diaminobenzidine (DAB). Quality control evaluation was performed on the stained slides to confirm correct and appropriate staining by comparison with controls. The IHC stained slides were reviewed and assessed using a semi-quantitative method to reveal the intensity and position of staining i.e. by the presence of granular staining in the glomeruli by the nephropathologist.

### Statistical analysis

Data analysis was performed using Stata 15.1 (StataCorp, TX, USA). Numerical variables were reported as mean (± standard deviation) if normally distributed and as median (interquartile range) if skewed. Analysis of variance (ANOVA), or its non-parametric equivalent (the Kruskall-Wallis test) was used to compare means across groups with pairwise comparisons performed using the Bonferroni correction. The Chi-square test was used to compare proportions. The sensitivity, specificity, positive predictive value (PPV) and negative predictive value (NPV), and their 95% confidence intervals, for diagnosis of PMN using positive PLA2R and THSD7A were calculated based on standard methods.[18] A p-value < 0.05 was taken as significant.

## Results

### Demographic and Clinical Characteristics

Overall, there were eighty-eight (88) patients included in this study: 41 with PMN, 19 with pure class V lupus nephritis (LN-V) and 28 with diabetic nephropathy (DN). Serologies for HIV, Hepatitis B and syphilis were negative for all study subjects. The mean age at presentation for patients with PMN was 44.5 ± 17.5 years; patients with LN-V were significantly younger (p = 0.02). There was an overall female preponderance, however, there was no significant gender difference observed across groups (p = 0.07). Patients with PMN had a significantly higher serum cholesterol (p = 0.001) and lower serum albumin (p = 0.004) while the eGFR was lowest in the group with DN (P = 0.001). Although the UPCR was much higher in the PMN group, there was no significant difference between groups (p = 0.07). Other clinical and biochemical results are shown in Table 1.

**Table 1:** Demographic and Clinical Characteristics of the study population

	PMN (*n* = 41)	LN-V (*n* = 19)	DN (*n* = 28)	*p*-value	Total (*n* = 88)
Age (years)	44.5 ± 17.5	33.8 ± 13.9	46.8 ± 14.2	0.02	42.9 ± 16.4
Female gender [n (%)]	25 (61.0)	17 (89.5)	18 (64.3)	0.07	60 (68.2)
Race (Black) [n (%)]	13 (31.7)	7 (36.8)	9 (32.1)	0.92	29 (32.9)
mABP (mmHg)	94.6 ± 11.7	85.7 ± 8.0	99.4 ± 16.1	0.01	94.3 ± 13.4
Presence of oedema [n (%)]	26 (89.7)	8 (53.3)	16 (64.0)	0.02	50 (72.5)
Hb (g/dL)	11.8 ± 2.1	10.6 ± 2.3	9.9 ± 1.7	0.001	11.0 ± 2.2
Platelet count (x10^9^/L)	368.4 ± 129.2	307.4 ± 149.4	340.9 ± 137.1	0.34	348.3 ± 135.7
WBC count (x10^9^/L)	8.4 ± 2.4	8.5 ± 4.6	9.0 ± 3.2	0.76	8.6 ± 3.1
Total cholesterol (mmol/L)	9.3 (7.8–11.6)	4.6 (3.7–5.8)	6.3 (4.8–7.9)	0.001	7.8 (5.5–9.6)
Serum albumin (g/L)	21.8 ± 7.1	25.4 ± 7.4	28.0 ± 5.9	0.004	24.3 ± 7.3
UPCR (g/mmol creatinine)	0.87 (0.49–1.26)	0.54 (0.27–0.75)	0.52 (0.17–1.34)	0.07	0.72 (0.34–1.24)
Serum creatinine (µmol/L)	80 (63–103)	72 (51–123)	188 (97–471)	0.001	96 (66–167)
eGFR (mL/min/1.73 m^2^)	85 (57–124)	98 (49–137)	30 911 − 63)	0.001	68 (38–111)

*PMN* Primary membranous nephropathy, *LN-V* class V lupus nephritis, *DN* diabetic nephropathy, *mABP* Mean Arterial Blood Pressure, *Hb* Haemoglobin, *WBC* White Blood Cell, *UPCR* Urine Protein Creatinine Ratio, *eGFR* estimated glomerular filtration rate

The 24-hour value of proteinuria (g/24Hrs) is approximately 10-times the ratio of the UPCR in g/mmol creatinine

### Histologic Characteristics

The histological features of included biopsies are summarized in Table 2. There were significant differences in the number of sclerosed glomeruli, presence of tubular atrophy and interstitial fibrosis (all highest in the DN group). PLA2R and THSD7A were only positive in the PMN group (51.2% and 4.9%, respectively) and were negative in the control groups. Only one patient with PMN showed enhanced granular expression of both PLA2R and THSD7A while another PMN patient who was negative for PLA2R was positive for THSD7A. A comparison of the demographic and clinical characteristics of PMN patients with positive and negative expression of PLA2R in their biopsies is shown in Table 3. There were no identified demographic or clinical differences between both groups.

**Table 2:** Histologic Characteristics of biopsies included in this study

	PMN (*n* = 41)	LN-V (*n* = 19)	DN (*n* = 28)	*p*-value	Total
Number of glomeruli per view (n [IQR])	15 (10–21)	16 (8–20)	16 (10–26)	0.47	15 (10–23)
Sclerosed glomeruli (n)	0.0 (0.0-6.7)	0.0 (0.0-2.6)	22.5 (8.4–48.7)	0.001	0.0 (0.0-14.8)
Tubular atrophy [n (%)]	7 (17.1)	4 (21.1)	18 (64.3)	< 0.001	29 (32.9)
Interstitial fibrosis [n (%)]	12 (29.3)	7 (36.8)	26 (92.9)	< 0.001	45 (51.1)
Immunohistochemistry:					
• IgG [n (%)]	31 (75.6)	16 (84.2)	4 (14.3)	< 0.001	51 (57.9)
• IgA [n (%)]	9 (21.9)	7 (36.8)	1 (3.6)	0.02	17 (19.3)
• IgM [n (%)]	16 (39.0)	13 (68.4)	8 (28.6)	0.02	37 (42.1)
• C3 [n (%)]	31 (75.6)	10 (52.6)	7 (25.0)	< 0.001	48 (54.5)
PLA2R (positive)	21 (51.2)	0 (0.0)	0 (0.0)	< 0.001	21 (23.9)
THSD7A (positive)	2 (4.9)	0 (0.0)	0 (0.0)	0.31	2 (2.3)

*PMN* Primary membranous nephropathy, LN-*V* class V lupus nephritis, *DN* diabetic nephropathy, *IQR* Interquartile range; *IgA* Immunoglobulin A, *IgG* Immunoglobulin G, *IgM* Immunoglobulin G, *C3* Complement factor 3; *PLA2R* phospholipase A-2 receptor; *THSD7A* thrombospondin type-1 domain containing 7A

**Table 3:** Characteristics of Patients with positive expression of PLA2R in the glomeruli

	PMN (PLA2R positive) (*n* = 21)	PMN (PLA2R negative) (*n *= 20)	*p*-value
Age (years)	44.71 ± 14.93	43.90 ± 20.46	0.88
Female gender [n (%)]	11 (52.38)	14 (70.00)	0.25
Race (Black) [n (%)	7 (33.33)	14 (66.67)	0.82
mABP (mmHg)	93.7 ± 10.8	95.48 ± 12.91	0.65
Presence of oedema (%)	12 (57.1)	14 (70.0)	0.39
Hb (g/dl)	12.1 ± 1.9	11.5 ± 2.2	0.35
Platelet count (x10^9^/L	363.3 ± 147.5	374.7 ± 106.4	0.79
WBC count (x10^9^/L)	7.8 ± 2.0	9.2 ± 2.7	0.06
Total cholesterol (mmol/L)	10.6 ± 4.2	8.9 ± 3.5	0.25
Serum albumin (g/L)	22.8 ± 6.3	20.5 ± 7.9	0.34
UPCR (g/mmol creatinine)	0.9 (0.7–1.1)	0.8 (0.4–1.3)	0.65
Serum creatinine (µmol/L)	88.0 (64.0–106.0)	77.5 (61.0–101.0)	0.50
eGFR (mL/min/1.73 m^2^)	85.0 (54.0–111.0)	85.5 (60.0–131.5)	0.57
Tubular atrophy [n (%)]	5 (23.8)	2 (10.0)	0.24
Interstitial fibrosis [n (%)]	5 (23.8)	7 (35.0)	0.43
IgG positive [n (%)]	18 (85.7)	13 (65.0)	0.12
IgA positive [n (%)]	5 (23.8)	4 (20.0)	0.77
IgM positive [n (%)]	11 (52.4)	5 (25.0)	0.07
C3 positive [n (%)]	17 (80.9)	14 (70.0)	0.41

*mABP* Mean Arterial Blood Pressure; *Hb* Haemoglobin; *WBC* White Blood Cell; *UPCR* Urine Protein Creatinine Ratio; *eGFR* estimated glomerular filtration rate; *IgA* Immunoglobulin A; *IgG* Immunoglobulin G; *IgM* Immunoglobulin M; *C3* Complement factor 3; *PLA2R* phospholipase A-2 receptor

The 24-hour value of proteinuria (g/24Hrs) is approximately 10-times the ratio of the UPCR in g/mmol creatinine

### Comparison of PLA2R positive PMN and class V LN

Table 4 shows the clinical and histological features of patients with positive PLA2R and LN-V group. Both serum cholesterol (p = 0.002) and UPCR (P = 0.04) were significantly higher in the positive PLA2R group. There were no significant differences in the histological features between both groups.

**Table 4:** Characteristics of Patients with positive expression of PLA2R and LN-V

	PMN (PLA2R positive) (*n* = 21)	LN-V (*n* = 19)	p
Age (years)	44.71	33.8 ± 13.9	0.02
Female gender [n (%)]	11 (52.4)	17 (89.5)	0.01
Race (Black) [n, %]	7 (33.3)	7 (36.8)	0.82
mABP (mmHg)	93.7 ± 10.8	85.7 ± 8.0	0.03
Presence of oedema [n (%)]	12 (57.14)	8 (42.11)	0.34
Hb (g/dL)	12.1 ± 1.9	10.6 ± 2.3	0.03
Platelet count (x10^9^/L)	363.3 ± 147.5	307.4 ± 149.5	0.28
WBC count (x10^9^/L)	7.8 ± 2.0	8.5 ± 4.6	0.53
Total cholesterol (mmol/L)	9.9 (8.7–11.8)	4.6 (3.7–5.8)	0.002
Serum albumin (g/L)	22.8 ± 6.28	25.45 ± 7.35	0.30
UPCR (g/mmol creatinine)	0.9 (0.7–1.1)	0.54 (0.3–0.8)	0.04
Serum creatinine (µmol/L)	88.0 (64.0–106.0)	72.0 (51.0–123.0)	0.65
eGFR (mL/min/1.73 m^2^)	85.0 (54.0–111.0)	98.0 (49.0–137.0)	0.74
Number of glomeruli per view (n)	14 (10–20)	16 (8–20)	0.97
Tubular atrophy [n (%)]	5 (23.8)	4 (21.1)	0.83
Interstitial fibrosis [n (%)]	5 (23.8)	7 (36.8)	0.37
Immunohistochemistry:			
• IgG [n (%)]	18 (85.7)	16 (84.2)	0.89
• IgA [n (%)]	5 (23.8)	7 (36.8)	0.37
• IgM [n (%)]	11 (52.4)	13 (68.4)	0.30
• C3 [n (%)]	17 (80.9)	10 (52.6)	0.06

*mABP* Mean Arterial Blood Pressure; *Hb* Haemoglobin; *WBC* White Blood Cell; *UPCR* Urine Protein Creatinine Ratio; *eGFR* estimated glomerular filtration rate; *IgA* Immunoglobulin A; *IgG* Immunoglobulin G; *IgM* Immunoglobulin M; *C3* Complement factor 3; *PLA2R* phospholipase A-2 receptor

The 24-hour value of proteinuria (g/24Hrs) is approximately 10-times the ratio of the UPCR in g/mmol creatinine

### Sensitivity and Specificity of PLA2R and THSD 7A for idiopathic membranous nephropathy

Table 5 provides a summary of the sensitivity, specificity, positive and negative predictive values of enhanced expression of PLA2R and THSD7A (alone and in combination) for predicting PMN. PLA2R and THSD7A (alone or when combined) showed a specificity and positive predictive values (PPV) of 100%. The sensitivity (alone or combined) of PLA2R and THSD7A were 51.2% [95%CI 35.1% − 67.1%], 4.9% [95%CI 0.6% − 16.5%] and 53.7% [95%CI 37.4% − 69.3%] respectively. There was no difference in the sensitivity and specificity when using PLA2R alone compared to combining the two tests (p = 0.32) (analysis not shown).

**Table 5:** Sensitivity, specificity, PPV and NPV of PLA2R and THSD7A

	Sensitivity	Specificity	PPV	NPV
**PLA2R**	51.2 (35.1–67.1)	100 (92.5–100)	100 (83.9–100)	96.7 (95.5–97.5)
**THSD7A**	4.9 (0.6–16.5)	100 (92.5–100)	100 (93.3–94.1)	93.7 (93.3–94.1)
**PLA2R & THSD7A**	53.7 (37.4–69.3)	100 (92.5–100)	100 (84.6–100)	71.2 (58.7–81.7)

All values are presented as percentage and (95% confidence interval); *PPV *positive predictive value; *NPV *Negative predictive value; *PLA2R* phospholipase A-2 receptor; *THSD7A* thrombospondin type-1 domain containing 7A

## Discussion

With the identification of candidate podocyte antigen targets such as PLA2R and THSD7A and their respective PLA2R and anti-THSD7A autoantibodies, the autoimmune basis of MGN has effectively been established. Our study aimed to determine the occurrence of novel antigens associated with MGN among South African patients previously defined as PMN by standard microscopic techniques. We found a respective frequency of occurrence of positive glomerular staining for PLA2R and THSD7A in 51.2% (21/41 patients) and 4.9% (2/41 patients) of biopsies initially categorized as PMN. None of the biopsies of LN-related MGN or DN stained positively for either PLA2R or THSD7A.

Since the canonical work of Beck et al. [7] in which the autoimmune target podocyte antigen PLA2R was discovered, the prevalence of PLA2R antibody in serum and/or PLA2R antigen in kidney tissue deposits in PMN have been variously described among different populations. In our cohort of predominantly non-black South Africans, the frequency of occurrence of histologic autoantibody positivity was relatively low at 51.2% compared to data from European cohorts in whom rates from 69.2–73.8% have been observed.[10, 19] Among Japanese patients however, lower rates as seen in our study have also been reported.[20] Evidence continues to accrue for a genetic component in the pathogenesis of PMN and a genetic role in the varying prevalence observed particularly with respect to PLA2R autoantibody positivity across different ethnicities. *HLA-DQA1* and *PLA2R1* are risk alleles for PMN that have been identified and demonstrated to confer different disease risk in different populations with a strong association shown among European, Indian, Chinese but not African Americans patients.[21–23].

Approximately 1 in 2 patient histologic samples initially identified as PMN by standard microscopic techniques did not demonstrate enhanced glomerular immune deposits staining for PLA2R antigen. This PLA2R immune deposit negative state could be accounted for by either of two possibilities. It is probable that these are indeed PMN cases with yet to be identified target podocyte antigen(s) as research continues to identify other candidate glomerular antigens that are autoimmune targets in PMN. Indeed 1 of our 20 PLA2R negative patients had a positive glomerular immune deposit staining for THSD7A, an antigenic target that was identified 6 years after PLA2R. Other novel proteins including neural epidermal growth factor-like 1 protein (NELL-1) [24] and Semaphorin 3B [25] have recently been identified to be associated with membranous nephropathy. NELL-1 was present in 23% [29/126] of European PLA2R and THSD7A negative PMN patients while Semaphorin 3B was predominantly present in paediatric patients with MGN.[24, 25].

These discoveries lend credence to our hypothesis that other glomerular basement membrane antigens may well underly the pathogenesis of PMN in this African cohort, especially with our relatively younger group of patients. An alternate explanation could be that these are secondary cases of MGN currently misidentified as PMN as predisposing factors such as in chronic viral infections like chronic hepatitis B, autoimmune disorders such as LN and solid organ malignancies were not clinically apparent and detectable by routine checks at the time of renal biopsy. Hepatitis-B associated glomerular disease and LN constitute the two commonest causes of secondary GNs in Africa and in both entities glomerular disease has been known to occur in the absence of systemic evidence of chronic hepatitis B antigenemia and lupus serologies respectively.[3, 26] We however could not demonstrate any significant clinical or histologic differences between our PLA2R positive and PLA2R negative patients to suggest that they represent two distinct pathogenetic groups of patients (Table 3). Unfortunately, we were unable to stain the biopsies for exostosin which can detect cases labelled as PMN but related to LN. Sethi et al.[27] detected exostosin 1 (EXT1) and exostosin 2 (EXT2) in 21 cases of PLA2R-negative MN, but not in PLA2R-associated MN and control cases, suggesting that subset of MN is associated with accumulation of EXT1 and EXT2 in the GBM. Thus, a yet to be identified glomerular antigen in this African group of PMN patient may be a more probable theory underlying PLA2R negative immune deposit staining. It is also possible, although we used an IHC method that has been previously described, that methodological differences in IHC techniques could have contributed to the proportion of PLA2R positive biopsies in our group.

Distinguishing between PMN and LN-V histologically has traditionally rested upon the additional demonstration of subendothelial deposits, mesangial hypercellularity and positive IHC/IF staining for C3, C1q, IgM, IgA and IgG1/IgG3 subclass in LN-V biopsies. Positive staining for glomerular PLA2R antigen by IF/IHC has now been added to the armamentarium of diagnostic tools in differentiating between PMN and LN-V with the latter typically, but not always, demonstrating negative serum PLA2R antibodies and/or glomerular staining for the PLA2R antigen.[28] Although we were not able to subtype IgG class or stain for C1q, we could not broadly exemplify this differential IHC staining pattern between our PMN patients and LN-V patients (Table 4). Although immunofluorescence examination can aid in differentiating LN-V from PMN given the absence of IgM, IgA, and C1q in the latter, it has been noted that these differences can be challenging due to the overlap that can occur.[20, 29] This may explain the presence, in a few PMN patients, of positive IgA, IgM and C1q despite these patients testing negative for all common secondary causes of MGN. Based on our findings there were notable clinical differences between PLA2R positive patients and LN-V patients with PLA2R patients having 1.7 fold heavier proteinuria and more than 2-fold higher total serum cholesterol levels than lupus MN patients; as is typical for the demographic epidemiology of lupus, there was a female predisposition for lupus. Dual positivity for the glomerular PLA2R and THSD7A antigens in patients with PMN is being increasingly reported from some studies.[30, 31] Only one patient in our cohort demonstrating dual positivity was demonstrated. It remains unclear the clinical implications of this dual staining pattern.

Our study is not without its limitations. Firstly, we were unable to stain the PLA2R negative samples for EXT1 and EXT 2, especially considering the younger age of patients in our study. This may have shown, as others have done in recent studies,[27] that some PLA2R negative patients treated as PMN could be patients with LN. Additionally, there was no outcome data reported for the patients to evaluate the role of glomerular PLA2R or THSD7A reactivity with response to therapy and disease progression. Our study was not designed to do this as we wanted to assess the relevance of these tests in an African population given that there are no previous studies. Furthermore, we were also unable to include the biopsies of patients with cancers and secondary MN to assess if THSD7A shows any association in such patients in our population. Lastly, we did not have serum samples at the time of diagnosis of MGN to assess serum PLA2R antibodies and determine if there existed any correlation between serum PLA2R antibodies and glomerular staining in our PLA2R positive patients. Despite these limitations and given the high specificity and PPV of our study results, it shows that glomerular PLA2R and THSD7A staining can be used for identifying PMN in African patients with MGN. We also encourage outcome studies using these tests in patients with biopsy proven PMN as this will improve the use of appropriate therapies for treating patients.

## Conclusions

This study shows that glomerular staining of PLA2R and THSD7A can be used to identify patients with PMN in South African patients with biopsy proven MGN. This means that immunotherapies for such patients can be appropriately initiated and judiciously monitored. Our study also showed that a combination of PLA2R and THSD7A may not provide better diagnostic accuracy compared to PLA2R only and that PMN patients with positive glomerular staining for PLA2R tend to have a more severe form nephrotic syndrome evidenced by higher UPCR compared with PLA2R-negative patients. There is need for further studies in other African populations to assess if glomerular and/or serum PLA2R and THSD7A can be used for identifying patients with PMN.

## Data Availability

The datasets used and analysed during the current study are available from the corresponding author on reasonable request.
